# Emerging Trends in Bilosomes as Therapeutic Drug Delivery Systems

**DOI:** 10.3390/pharmaceutics16060697

**Published:** 2024-05-23

**Authors:** Hemlata Kaurav, Meenakshi Tripathi, Simran Deep Kaur, Amit Bansal, Deepak N. Kapoor, Sandeep Sheth

**Affiliations:** 1 School of Pharmaceutical Sciences, Shoolini University of Biotechnology and Management Sciences, P.O. Box 9, Solan 173229, Himachal Pradesh, India; kaurav.hem88@gmail.com (H.K.); meenakshitripathi15012000@gmail.com (M.T.); deepakpharmatech@gmail.com (D.N.K.); 2 Formulation Research and Development, Perrigo Company plc, Allegan, MI 49010, USA; amitbansal2010@gmail.com; 3 Department of Pharmaceutical Sciences, College of Pharmacy, Larkin University, Miami, FL 33169, USA

**Keywords:** bilosomes, bile salts, drug delivery, topical delivery, ultra-deformable vesicles

## Abstract

In recent years, there has been a notable surge in the utilization of stabilized bile acid liposomes, chemical conjugates, complexes, mixed micelles, and other drug delivery systems derived from bile acids, often referred to as bilosomes. The molecular structure and interactions of these amphiphilic compounds provide a distinctive and captivating subject for investigation. The enhanced stability of new generation bilosomes inside the gastrointestinal system results in the prevention of drug degradation and an improvement in mucosal penetration. These characteristics render bilosomes to be a prospective nanocarrier for pharmaceutical administration, prompting researchers to investigate their potential in other domains. This review paper discusses bilosomes that have emerged as a viable modality in the realm of drug delivery and have significant promise for use across several domains. Moreover, this underscores the need for additional investigation and advancement in order to comprehensively comprehend the prospective uses of bilosomes and their effectiveness in the field of pharmaceutical administration. This review study explores the current scholarly attention on bilosomes as prospective carriers for drug delivery. Therapeutic areas where bilosomes have shown outstanding performance in terms of drug delivery are outlined in the graphical abstract.

## 1. Introduction

New approaches in drug development have resulted in the introduction of several compounds with subpar bioavailability profiles. Lipophilicity is a problem for many therapeutic compounds because it reduces their solubility in gastrointestinal (GI) fluids and hence their bioavailability [1]. Most biological molecules (proteins and peptides) and vaccines do not work well when taken orally because they degrade extensively in the digestive tract. Numerous strategies have been implemented to address the shortcomings of conventional delivery methods. Vesicular carriers like liposomes and niosomes, which are part of the innovative drug delivery system, can protect the encapsulated components from degradation to some degree. Bilosomes are the novel modified form of liposomes and niosomes with the advantage of more flexibility, absorption, and permeation due to the presence of bile salts. Bilosomes are bile salt-containing nano vesicular systems used for the administration of highly lipophilic and poorly permeable therapeutics [2,3]. The role of bile acids in drug delivery as permeation enhancers is well explained in the literature [4,5,6]. Oral drug administration as an alternative to injectable therapy is made possible by bilosomes that protect therapeutic agents from being broken down in the stomach. Due to their stability in the gut, bilosomes improve the biocompatibility of medications and boost their therapeutic effectiveness [7]. Bilosomes are reported to improve the GI tract stability and permeability of hydrophilic molecules like proteins, peptides, and antigens across the cell membrane [8,9]. Due to their amphiphilic nature, bile salts incorporated within bilosomes, such as sodium glycocholate, sodium deoxycholate, and sodium taurocholate, enhance the penetration properties of the system, further improving the oral bioavailability and biodistribution of drugs with limited aqueous solubility and permeability [6,10].

Comparisons between conventional liposomes and niosomes formulations with advanced bilosomal formulations have demonstrated enhanced bioavailability and the permeation properties of bilosomes over other vesicular systems [11,12,13,14]. Liposomes and niosomes are both types of vesicular drug delivery systems, but they differ in their composition, structure, and properties. Liposomes are composed of phospholipids, which are natural or synthetic amphiphilic molecules with a hydrophilic head and hydrophobic tail. These phospholipids self-assemble into bilayer structures in aqueous environments, forming vesicles with aqueous cores enclosed by lipid bilayers. Niosomes, on the other hand, are composed of non-ionic surfactants, which are amphiphilic molecules similar to phospholipids but lack the phosphate group. Non-ionic surfactants typically consist of a hydrophilic head (such as polyethylene glycol) and a hydrophobic tail (such as alkyl chains). Niosomes also form vesicular structures similar to liposomes, with aqueous cores enclosed by surfactant bilayers. Liposomes formed by phospholipids tend to be more stable due to the presence of natural phospholipids with well-defined structures and packing arrangements. However, they may be susceptible to degradation by enzymes in biological fluids. Niosomes are generally less stable compared to liposomes, primarily because non-ionic surfactants may undergo phase transitions or degradation under certain conditions.

The therapeutic application of bilosomes as drug delivery carriers is well established for phytoconstituent drug delivery, anticancer drugs, CNS drugs, protein/peptide, and vaccine drug delivery [1,15,16,17]. In this review, the structural aspects and therapeutic application of bilosomes as nanovesicular drug delivery carriers are discussed. Moreover, a brief overview of bilosomes-based drug delivery systems for treating various disorders through different routes mainly oral, ocular, and transdermal are also discussed.

## 2. Methodology

In this review, we have summarized the advances in the application of bilosomes as a nanocarrier for drug delivery mainly for oral vaccines, proteins and peptides, and drugs used to treat CNS disorders, ocular disorders, and cancer. A literature search of relevant publications published in the last 10 years was performed using several scientific databases such as PubMed, Scopus, Elsevier, and ScienceDirect. The main keywords used to search the literature are bile salts, bilosomes, permeation enhancers, modified liposomes or niosomes, and ultra-deformable nanovesicles. Only English-language publications were included in this review.

## 3. Bilosomes: Structural Aspects

**Figure 1:**
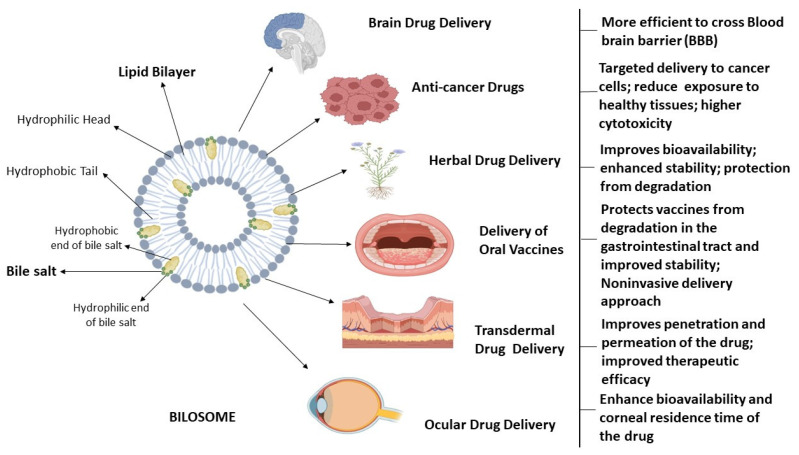
Bilosomes in therapeutic drug delivery. This is a schematic representation of the architectural characteristics of bilosomes, which are enclosed double-layered structures composed of non-ionic amphiphiles that consist of hydrophilic head and hydrophobic tails, as well as bile salts. Bilosomes serve as highly efficient nanocarriers for delivering medications to address a range of brain illnesses, targeted drug delivery for cancer treatment, ophthalmic ailments, and improving the absorption of orally and transdermally administered pharmaceuticals.

Conacher et al. were the first to describe bilosomes, which are closed bilayer structures of non-ionic amphiphiles that are structurally similar to non-ionic surfactant vesicles (niosomes) but additionally contain bile salts [10]. Bile salts shield the bilosomes and their contents from the acidic and alkaline conditions of the GI tract. Additionally, the inclusion of bile salts in bilosomal encapsulation has been shown to boost intestinal permeability, leading to the increased bioavailability of orally administered medications. Non-ionic surfactants, such as those found in the Span family (Span 40, Span 60, Span 80), are often used in the formulation of bilosomes, usually in conjunction with cholesterol and bile salts [1,3,18]. The components used in the formulation of bilosomes are widely acknowledged as safe and have been shown to exhibit perfect biocompatibility and biodegradability. Bilosomes, as a synthetic nano-sized vesicular carrier system, are a promising avenue for enhancing the efficacy of oral medication delivery across a diverse spectrum of pharmaceutical compounds. These vesicular nanostructures were analyzed for their ability to enclose hydrophilic and hydrophobic substances in their aqueous core and phospholipid bilayer, respectively. Bile salts, non-ionic surfactants, and lipids are principal vesicular components in the synthesis of bilosomes [19]. The bilosomes have two layers, with the innermost layer containing hydrophilic medicines and antigens and the outermost layer containing bile salts and hydrophobic medications. The bile salts are encased in lipid layers within the bilosomal structure, giving it a characteristic closed shape. The hydrophilic end of bile acid molecule is faced towards the hydrophilic part in the lipid bilayer whereas the hydrophobic end of bile acid is embedded in the hydrophobic part of lipid bilayer in a bilosome vesicle [2,20,21]. The structure of bilosomes and their utility in administering different types of drugs through different routes is illustrated in Figure 1.

### 3.1. Lipids as a Component of Bilosomes

Mostly, phospholipids and cholesterol are employed in the manufacturing of bilosomes. Phospholipids have a high degree of biocompatibility with biological membranes. The amphiphilic characteristic of phospholipids gives them the ability to self-assemble into concentric bilayers, resulting in wetting and emulsification [6]. The phospholipids often used in the formulation of bilosomes are dicetyl phosphate, distearoyl phosphatidylglycerol, soybean phosphatidylcholine, and monopalmitoyl glycerol [22]. Cholesterol is introduced into the cellular membrane as an amphiphilic molecule, with the hydroxyl groups towards the aqueous surface and the aliphatic chains aligned perpendicular to the acyl chains in the bilayer’s core. The effect of cholesterol on the bilosome membrane is dependent on the phase state of the lipid bilayer, which can be determined by the lipid composition. Cholesterol causes phase separations in multi-component lipid mixtures, selectively partitions between distinct coexisting lipid phases, and drives integral membrane proteins to change their conformation or to redistribute in the membrane [23]. Therefore, the rigidity of bilosomes is enhanced by adding cholesterol [24,25,26].

### 3.2. Non-Ionic Surfactant as a Component of Bilosomes

**Figure 2:**
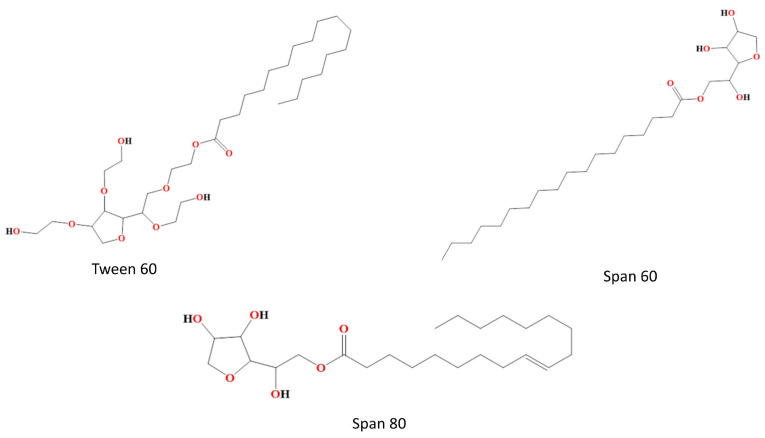
Non-ionic surfactants used in bilosome formulation, e.g., Tween 60, Span 60 and Span 80.

Non-ionic surfactants are often utilized in the formulation of bilosomes due to their superior stability and compatibility as compared to anionic, cationic, and amphoteric surfactants. Non-ionic surfactants are less hemolytic and more conducive in preserving the solution’s pH around its physiological range. These substances serve as solubilizers, wetting agents, emulsifiers, and permeability enhancers [27]. Additionally, non-ionic surfactants serve as potent inhibitors of P-glycoprotein, preventing drug efflux from cells. They facilitate the absorption of drugs and enable targeted delivery to certain tissues. Non-ionic surfactants consist of both polar and non-polar regions, hence exhibiting significant interfacial activity. The entrapment effectiveness of the medication is influenced by the chain length and size of the hydrophilic head groups of non-ionic surfactants. The entrapment efficiency of non-ionic surfactants, namely those containing stearyl (C18) chains, is greater compared to those containing lauryl (C12) chains [28,29]. To achieve the enhanced entrapment efficiency of water-soluble medications, a mixture of Tweens including elongated alkyl chains and substantial hydrophilic components, combined with cholesterol in a 1:1 ratio, can be used [30]. The hydrophilic–lipophilic balance (HLB) value of surfactants is a significant factor in the regulation of drug entrapment inside vesicles. Surfactants with an HLB within the range of 14–17 have been deemed unsuitable for the production of bilosomes vesicles. Conversely, an HLB value of 8.6 has been shown to provide maximum effectiveness in terms of entrapment. The entrapment efficiency decreases when the HLB value decreases from 8.6 to 1.7. The non-ionic surfactants that are often used for the purpose of vesicle production are alkyl esters, alkyl glyceryl ethers and sorbitan fatty acid esters [30]. Non-ionic surfactants which are mainly used in bilosome formulation are shown in Figure 2.

### 3.3. Bile Salts as a Component of Bilosomes

Bile salts are endogenous biosurfactants that are found inside the lumen of the gastrointestinal tract. They serve a crucial function in facilitating the digestion and absorption of lipids. Increased bile production results in the absorption of more physiologically active chemicals. This has led to the development of several mixed micelle systems to enhance the solubility of extremely lipophilic pharmaceuticals [31]. Bile salts have the additional effect of improving the flexibility of bilosomes in simulated fluids and the same is reported by Mondal et al. through the use of microscopy and fluorescence techniques [32]. This is achieved through the induction of repulsion between the bile salts contained inside the bilosomes and the external bile salts found in the gut lumen. The most common bile salts used in the manufacturing of bilosomes are sodium glycocholate (SGC), sodium deoxycholate (SDC), and sodium taurocholate (STC). The selection of sodium glycocholate (SGC), sodium deoxycholate (SDC), and sodium taurocholate (STC) as the main bile salts in bilosomes is primarily due to their unique properties and advantages in the drug delivery system [29,30]. These bile salts possess excellent solubilization properties, particularly for lipophilic compounds. By forming mixed micelles with lipids, they can encapsulate hydrophobic drugs effectively, enhancing their solubility in aqueous environments. This is crucial for drug delivery systems like bilosomes, where the efficient solubilization of drugs is essential for their bioavailability and therapeutic efficacy. SGC, SDC, and STC are naturally occurring bile salts found in the bile of mammals, including humans. They are biocompatible and well-tolerated by the body, which is essential for pharmaceutical applications. Their use minimizes the risk of adverse effects, making them suitable for incorporation into drug delivery systems intended for medical use. These salts can interact with cell membranes, facilitating the absorption of encapsulated drugs. This property is advantageous for enhancing the delivery of therapeutic agents to target cells or tissues. By incorporating bile salts with a lipid membrane, the bioavailability and therapeutic outcomes of the encapsulated drugs in bilosomes can be improved. These salts contribute to the stability of bilosomes by participating in the formation of bilayer structures alongside phospholipids. This helps to prevent the aggregation or fusion of bilosomes, ensuring their integrity during storage and administration. Overall, the selection of SGC, SDC, and STC as the primary bile salts in bilosomes is based on their solubilization ability, biocompatibility, interaction with cell membranes, and contribution to structural stability, all of which are crucial for the successful formulation and performance of bilosomes as drug delivery system [20].

## 4. Bilosomes as Nanocarrier Drug Delivery System for Treating Various Disorders

Bilosomes are a novel and promising lipid-based nanocarrier system used in pharmaceutical drug delivery. These nanocarriers have gained attention due to their potential to enhance the delivery and bioavailability of drugs, particularly for both hydrophilic and lipophilic compounds. Bilosomes as a nanocarrier drug delivery system for treating various disorders are discussed below.

### 4.1. Bilosomes as Drug Delivery System for Brain Disorders

The blood–brain barrier (BBB) is the primary stumbling block in the way of novel therapies for entering the central nervous system [33,34]. The BBB is composed of three biological components of the brain microvasculature: endothelial cells, astrocyte end-feet, and pericytes [35]. Since bile acids are orally bioavailable and may cross the microvascular endothelial cells in the human brain, studying their role in neurodegenerative diseases is an exciting prospect [36,37]. CNS drugs with limited therapeutic efficacy can be encapsulated into bilosomes for brain targeting. The bioavailability of such formulations could be enhanced by administering them through an oral or intranasal route.

For instance, Abbas et al. [38] developed resveratrol (RES)-loaded bilosomes for the management of Alzheimer’s disease (AD) through the oral route of administration. Resveratrol (RES), a well-known bioactive polyphenol compound, is used to treat AD but its clinical applicability is limited due to poor water solubility [39]. In vitro drug release showed 97% of the RES released from RES-loaded bilosomes as compared to the 25% of the drug released from RES suspension after 2 h [38]. In the mice model of AD, RES-loaded bilosomes outperformed the standard drug suspension in improving mice memory using the Y-maze and Morri’s water maze tests. In another study by the same group, the brain targeting of RES bilosomes through the intranasal route was investigated. In this study, the formulation of RES-loaded and chitosan (CS)-coated bilosomes was developed, which was compared with SPION (superparamagnetic iron oxide nanoparticles)-loaded CS-coated bilosomes to allow bilosomes guidance and retention in the brain by the use of an external magnetic field [40]. An in vivo evaluation in a lipopolysaccharide-induced AD mice model demonstrated that the CS-coated SPION bilosomes outperformed in reducing neuroinflammatory markers and improving cognitive and memory functions as compared to the CS-coated bilosomes and RES suspension. Conclusively, in vitro and in vivo data have suggested that the therapeutic efficacy of the poorly bioavailable neuroprotective drug RES can be improved by encapsulating it in bilosomes and this could be a viable medication delivery approach for the treatment of AD [38,40].

Another drug, zolmitriptan, belongs to the third class of the biopharmaceutics classification system (BCS) due to its high solubility and low permeability. Taweel et al. [41] developed zolmitriptan-loaded bilosomes and encased them in a mucoadhesive in situ gel with a delayed nasal mucociliary transit time so that the zolmitriptan could be delivered intranasally to the brain. With a sol–gel temperature of 34.03 °C, the bilosomal formulation of the mucoadhesive in situ gel enhanced the mucociliary transit time by 22.36 min. The intranasal distribution through the gelling system resulted in greater brain bioavailability than bilosomal dispersion [41]. Elsheikh et al. [42] developed intranasally administered luteolin-loaded bilosomes for use in preserving both short- and long-term spatial memory in AD patients. Luteolin is a medicine of dubious efficacy due to its poor solubility. Formulation evaluation results revealed an entrapment efficiency of 70.4% and a maximum in vitro drug release of up to 98.7 ± 4.20% from luteolin-loaded bilosomes as compared to 9.75 ± 1.01% from a pure drug suspension. In vivo findings in a sporadic AD mouse model demonstrated that luteolin-loaded bilosomes boosted both short- and long-term spatial memory by suppressing amyloid β aggregation and hyperphosphorylated Tau protein levels in the hippocampus [42]. In a recent study, an herbal extract of *Bacopa monnieri*, which is reported to possess strong neuroprotective activities, was encapsulated into liposomes and bilosomes to improve its oral bioavailability. In vitro and in vivo evaluation results for both formulations showed the better potential of *B. monnieri*-loaded bilosomes in enhancing bioavailability as compared to *B. monnieri*-loaded liposomes [43].

Numerous methods have been investigated and different drug formulations have been proposed in an attempt to enable greater drug penetration into the brain despite the BBB continuing to be a considerable barrier for pharmaceuticals to pass. Since bilosomes are permeation-modifying agents and drug absorption enhancers, they merit more research for application in brain drug delivery in the future.

### 4.2. Bilosomes as an Ocular Drug Delivery System

Novel therapeutics for the treatment of ocular disease have been developed as a result of recent advancements in drug delivery techniques and materials sciences. Currently, only topical application, redistribution into the eye after systemic administration, or targeted intraocular/periocular injections can deliver drugs to the eye [44]. Drug transport to the targeted ocular tissues is impeded by a number of precorneal, dynamic, and static ocular obstacles. The conventional system for ocular drug delivery suffers with the problem of poor bioavailability due to low corneal residence time and the fast elimination of the administered dose from the nasolacrimal drainage system [45]. Moreover, the target tissues do not retain therapeutic medication levels for a prolonged period of time. As the eye is a sensitive and delicate human organ, the adverse effect is a major concern while designing the drug delivery system for ocular diseases [46]. To overcome these limitations and to improve ocular delivery including delivery to the anterior segment, topical gelling systems are being explored. The incorporation of drug-loaded bilosomes into an in situ gelling system is reported to be advantageous over conventional modes of drug delivery for treating ocular disorders in terms of increased transcorneal flux, improved ocular residence time, controlled drug release, increased bioavailability, and ocular permeability.

Abdelbary et al. [47] employed ultra-deformable bilosomes to encapsulate the water-insoluble antifungal medication terconazole for ocular delivery. In ex vivo studies, drug flux through a rabbit cornea was found to be enhanced by the optimal ultra-deformable bilosomes formulation compared to that of conventional bilosomes, niosomes, and drug suspension [47]. In another study, Janga et al. [48] tested the ocular delivery of bilosomes loaded with the ion-sensitive in situ gel of natamycin, an antifungal agent. The transcorneal flux of the natamycin-loaded bilosomes (NB) increased six to nine times, indicating that bilosomes were effectively absorbed by the eyes. Corneal histology and in vitro cytotoxicity tests showed that the natamycin-loaded bilosomes were safe and cytocompatible for ocular application. The results demonstrated that ion-sensitive in situ hydrogels loaded with bilosomes provided an efficient platform for ocular medication that lasted longer and normalised natamycin levels in rabbit eye tissues [48].

Several medications used for the treatment of glaucoma have been formulated in bilosomes to test their effectiveness through ocular delivery. Acetazolamide (ACZ), which is used to treat glaucoma, suffers from poor corneal penetration due to low solubility and permeability. Mohsen et al. [49] encapsulated acetazolamide in bilosomes to test its ocular delivery through the cornea. In vitro data for ACZ-loaded bilosomes showed the highest entrapment efficiency of 74.24% and biphasic drug release pattern starting in 30 min followed by a slow and prolonged release for up to 8 h. In vivo pharmacokinetic and pharmacodynamic studies in male albino rabbits revealed the highest bioavailability from the bilosomal formulation as compared to ACZ niosomes and marketed ACZ formulations, including plain ACZ, ACZ oral tablets, and dorzolamide eye drops. Moreover, the ACZ-loaded vesicular formulations demonstrated a slower decrease in intraocular pressure which lasted for 1 h. Lastly, the Draize irritancy test confirmed that the ACZ bilosomal formulation was safe after topical application to the eye. Therefore, the bilosomal formulation is thought to be a potentially effective nanocarrier for the administration of acetazolamide to the eyes in order to treat increased intraocular pressure [49]. Nemr et al. [50] developed AGO-loaded hyaluronic acid-bilosomes using D-optimal design to enhance its bioavailability in ocular tissues. An in vitro drug release study revealed a slow and controlled drug release from all bilosomal formulations which lasted for 24 h as compared to the AGO solution which released most of the drug within the first 2 h. Consequently, the AGO bilosomal formulation significantly reduced the intraocular pressure in male albino rabbits when compared to its solution. Histopathological evaluation showed no eye irritation or obscured vision with AGO bilosomal formulation, confirming its safety for topical application to the eye [50]. Sakr et al. [51] encapsulated betaxolol hydrochloride, a poorly bioavailable drug, into highly permeable bilosomes for ocular delivery for the treatment of glaucoma. In vitro drug release studies exhibited a biphasic and sustained release of most of the drug over 24 h period. An ex vivo permeation study showed a 2.2-fold increase in drug permeation compared to the conventional marketed dosage form. A preclinical assessment in male albino rabbits displayed 57.81% reduction in intraocular pressure compared to 35.27% in the case of the marketed dosage form, confirming improved transcorneal permeation [51].

Bilosomes loaded with antibiotics have been tested for the management of bacterial eye infections. Alsaidan et al. [52] developed and evaluated ciprofloxacin-loaded bilosomal in situ gel (CIP-BLO) for the management of bacterial conjunctivitis. The optimized CIP-BLO formulation showed sustained CIP release with superior entrapment efficiency. This formulation also exhibited > 2-fold permeability than pure CIP. Evidently, no sign of toxicity was displayed by the CIP-BLO formulation in a corneal hydration test (76.15%), histology, and the hen’s egg-chorioallantoic membrane (HET-CAM) test (zero scores). The antimicrobial activity against P. aeruginosa and S. aureus was also found to be significantly higher for CIP-BLO formulation than pure CIP. Conclusively, CIP-BLO was found to be an effective strategy for enhancing corneal residence time and the pharmacological activity of CIP [52]. Similarly, moxifloxacin (MX), another antibiotic used to treat conjunctivitis, was loaded into bilosomal in situ gel to improve ocular residence time. The bilosomal formulation of this antibiotic showed 2.8-fold higher permeation compared to pure MX solution [53].

**Figure 3:**
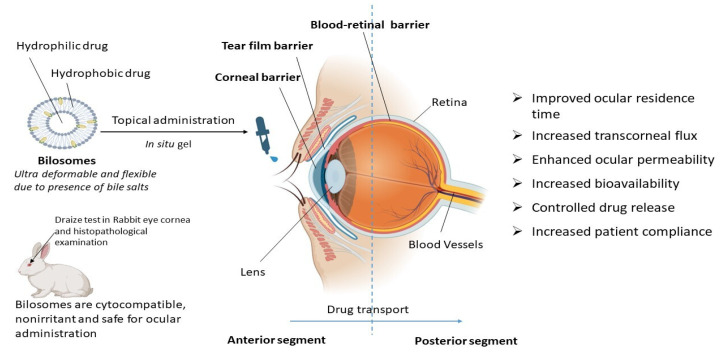
Therapeutic advantages of bilosomes in ocular drug delivery. Bilosomes are ultra-deformable novel vesicular carriers that are also used for ocular drug delivery. They are advantageous over conventional drug delivery systems for topical ocular administration in terms of controlled drug release, increased bioavailability, an improvement in ocular residence time, transcorneal flux, ocular permeability, and patient compliance.

From all these reported studies it can be concluded that bilosomes are efficient in ocular drug delivery due to increased transcorneal flux, prolonged corneal contact time, controlled ocular delivery, and improved bioavailability due to the ultra-deformable and flexible properties of bilosomes, as depicted in Figure 3. Bilosomal formulations are also reported to be safe and non-irritant for ocular administration [49,50]. Using the traditional ocular delivery system, the distribution of the drug in the target tissue is limited and its therapeutic levels are rapidly lost due to several precorneal, dynamic, and static barriers. In this regard, bilosomal formulations open up a huge possibility as a means of improving and extending eye care treatment.

### 4.3. Bilosomes for Delivery of Oral Vaccines

Oral dosing is the gold standard for patient compliance when it comes to the administration of vaccines. To elicit an immune response, the vaccine antigen is taken up by gut-associated lymphoid tissue consisting of Peyer’s patches overlying M cells. Antigens are then trafficked to dendritic cells, where they undergo a series of events that lead to the generation of mucosal and systemic immune responses. Although highly preferred, the oral route is not the recommended route of administration for most vaccines and biologicals due to poor absorption. Orally administered vaccines are entrapped in the mucus gel of GI tract epithelia and become diluted, denatured by enzymes, and excluded by an epithelial barrier, which leads to a weaker immune response. There is an unmet need for a delivery system that can withstand the degradation in the stomach and intestine. In this respect, bilosomes were found to be much more stable in the stomach’s acid and enzymes [2,54]. Moreover, due to the presence of bile salts, bilosomes can destabilize the membranes and were found to be an effective formulation for oral therapeutic delivery [1].

Conacher et al. [10] studied the efficacy of a bilosomes-encapsulating influenza subunit vaccine in mice and found a comparable antibody response to a parenterally administered vaccine using the same quantity of antigen. Moreover, the Th1/Th2 response was found to be similar following immunization by both routes of administration [10]. In another study, the conjugation of a cholera toxin B subunit (CTB) to hepatitis B surface antigen (HBsAg)-loaded bilosomes facilitated the binding of bilosomes to a Glucomannan-modified (GM)1 ganglioside receptor on M cells. The results showed the generation of comparable anti-HBsAg IgG antibody titer in mice when administered via an oral and intramuscular route [55]. However, the dose required via oral route is twice the dose required in intramuscular administration. Premanand et al. [13] investigated the efficacy of orally administered recombinant baculovirus (Bac-VP1) and bilosomes loaded with Bac-VP1 in a murine model for developing an antiviral vaccine against human enterovirus 71 (HEV71). It was found that Bac-VP1, associated with bilosomes, elicited significantly higher immune responses (both systemic and mucosal) compared to bilosomes administered alone. Interestingly, it was found that mice immunized subcutaneously with live Bac-VP1 had considerably greater VP1 specific blood IgG levels and stronger neutralizing antibody titers than mice inoculated orally with live Bac-VP1 alone or in combination with bilosomes [13]. Similarly, Wilkhu et al. [30] investigated the targeting efficacy of bilosomes containing the influenza vaccine to Peyer’s patches in mice. Their study results showed increased bilosomes in Peyer’s patches relative to a free antigen. Further studies were performed to evaluate the efficacy by challenging the mice with empty bilosomes and bilosomes containing the influenza antigen. Their study findings revealed a decrease in median body temperature and reduced viral load in the nasal secretion in a group of mice that received bilosomes containing influenza antigen compared to empty bilosomes [30]. In another study, the augmentation of immune response by targeting the M cells was investigated by Jain et al. [56] in which GM bilosomes loaded with tetanus toxoid (TT) undergo enhanced uptake through the mannosyl receptors of dendritic cells. The presence of abundant mannose molecules on GM-modified bilosomes facilitated the interaction with mannose receptors on dendritic cells, leading to enhanced cellular uptake. This resulted in an increased systemic immune response relative to conventional liposomes and additionally, mucosal immune response was also obtained via oral route [56].

Limited studies have been reported on delivering vaccines in conjugation with bilosomes through the oral route. Bilosomes could be useful in delivering vaccines through the oral route as they are noninvasive, painless, and effective in stimulating the systemic immune response against an antigen in the body. In this context, more research is needed to explore the potential of a bilosomes drug delivery system for the delivery of vaccines through the oral route of administration.

### 4.4. Bilosomes for Delivery of Proteins and Peptides

Proteins and peptides provide a potent therapeutic alternative to conventional small molecules for several diseases. Despite the superior therapeutic efficacy of large molecules when administered via the parenteral route, they have the limitations of a short biological half-life, poor patient compliance, and a need for technical skills for their administration. Oral administration of protein and peptide with a molecular weight above 700 Da showed poor stability in the GIT environment and had low permeation through the intestinal barrier, leading to a poor bioavailability of less than 10%. The limitation in the delivery of protein and peptides can be addressed using a vesicular nanocarrier, i.e., liposomes or niosomes, that can deliver both hydrophilic and hydrophobic molecules. However, there are reports of instability, leakage, and poor permeability of vesicular nanocarrier in the GI environment. Furthermore, gastric leakage exposes the peptides to GI enzymes, causing their denaturation [57]. Thus, the need for an alternative dosage form that can mitigate the issues associated with vesicular nanocarriers led to the development of a modified vesicular system called bilosomes. In bilosomes, bile salts are added to the bilayer thus stabilizing the vesicular nanocarrier and preventing leakage in the gastric milieu. The addition of bile salts in the bilayer also leads to its increased fluidity and thus improves permeability across GI membrane [5].

A study by Hu et al. [58] compared conventional liposomes and liposomes containing bile salts (sodium glycocholate) in their ability to enclose insulin and prevent gastric leakage. The testing was performed using simulated and ex vivo GI fluids. The results of the study demonstrated that liposomes made with bile salts conferred better protection and less gastric leakage relative to conventional liposomes [58]. A study by Niu et al. [17] investigated the bioavailability of insulin administered via oral route using liposomes containing bile salts in rats. In this study, the performance of conventional liposomes was compared with liposomes prepared using different bile salts (sodium deoxycholate (SDC), sodium taurocholate (STC), and sodium glycocholate (SGC). The study findings revealed that SGC liposomes showed higher bioavailability than SDC and STC liposomes. Insulin protection against gastric enzymes (pepsin, trypsin, and α-chymotrypsin) was also evaluated in vitro and the results obtained showed the superior protection of insulin against enzymatic degradation by liposomes containing bile salts compared to conventional liposomes [17]. Another study by the same research group investigated the ability of liposomes containing bile salts (BS liposomes) in enhancing the GI stability of insulin by using fluorescent imaging tools. They also studied the interaction of BS liposomes with the biomembrane using Caco-2 cell lines as a surrogate. The study results found that BS liposomes showed prolonged GI residence time and enhanced permeation compared to conventional liposomes. Visualization using confocal laser scanning microscopy further revealed trans-enterocytic internalization as a proposed mechanism of transport for BS liposomes across the biomembrane [59]. In another study, Nallamothu et al. [60] investigated the effect of the charge and chain length of bile salts in bilosomes on the oral bioavailability of insulin. Deoxycholic acid bile salts conjugated to amino acid were used and uptake studies were performed using the Caco-2 cell line. The study results revealed that anionic bilosomes were 4-fold more efficiently taken up relative to cationic liposomes (1.3-fold). The study results also demonstrated a 1.6-fold increase in AUC with bilosomes administered via the oral route relative to the subcutaneous route [60].

### 4.5. Bilosomes as Transdermal Drug Delivery System

Outer skin is the primary obstacle between the organism’s body and its outer environment. Stratum corneum, the epidermis’s outermost layer, is responsible for the skin’s excellent barrier characteristics [61]. Technological advances have made it feasible to transdermally distribute a broader spectrum of drugs, typically from hydrophobic small-molecule pharmaceuticals to hydrophilic medications and macromolecules [62]. Among novel drug delivery approaches for transdermal drug delivery, liposomes and niosomes are well explored. Later, the permeation characteristics of liposomes were improved by incorporating bile salts in them. Bile salts aid in the transportation of drugs across biological membranes either by enhancing drug dissolution or modifying cellular membrane permeability [20]. Bile salts also increase the flexibility of these nanovesicles which also leads to high transdermal permeability. In the literature, many reports are available showing the enhanced transdermal permeability of poorly soluble and bioavailable drugs utilizing bilosomes as a novel carrier for topical delivery, as shown in Table 1. Aziz et al. [11] altered the composition of traditional niosomes by adding various bile salts and then tested their efficacy in the transdermal distribution of diacerein (DCN). DCN is a poorly bioavailable (35–56%) osteoarthritic drug which has GI side effects when administered orally [63]. To establish its safety for topical usage, bilosomes were developed using factorial design optimization techniques. Optimized bilosome formulation displayed nanosized vesicles (301.65 ± 17.32 nm) and 100.00 ± 0.00% entrapment efficiency. Ex vivo permeation and in vivo studies demonstrated a higher permeation and an improved drug retention capacity of bilosomal formulation compared to the drug suspension and niosomal formulation. These findings corroborated the hypothesis that bilosomes are superior than niosomes for enhancing DCN permeation through the skin [11].

Another drug, olmesartan medoxomil (OLM), which is used as an antihypertensive agent, has a poor oral bioavailability of 26% which limits its therapeutic efficacy. So, to enhance its bioavailability, Albash et al. [24] developed and characterized olmesartan medoxomil-loaded PEGylated bilosomes for transdermal delivery. The higher deposition of OLM in rat skin was observed from bilosomal formulation compared to transethosomes and OLM suspension as per in vivo skin deposition studies. The optimized bilosomal formulation showed a higher relative bioavailability of 235.04% in the pharmacokinetic study performed in male albino rabbits, which is significantly higher than oral tablets [24]. Tizanidine hydrochloride (TZN), a muscle relaxant with poor oral bioavailability, was also formulated into bilosomes for topical delivery. Preclinical studies reported an improved transdermal permeation of the drug through bilosomal nanovesicles compared to the plain drug [64]. Similarly, Terbutaline (TBN) sulfate, which is used for the treatment of asthma and suffers poor oral bioavailability due to hepatic first-pass metabolism, was loaded into bilosomal gel formulation [65]. TBN chitosan-coated bilosomes (TBN-CTS-BLS) were optimized using a central composite design. A pharmacokinetic study in the rat model showed that the TBN bioavailability was increased by approximately 2.33-fold and t_1/2_ was increased to 6.21 ± 0.24 h when using the optimized TBN-CTS-BLS formulation compared to the oral TBN solution. This research revealed that BLS may be used as an effective and secure transdermal carrier for TBN to overcome hepatic first-pass metabolism [65]. Lornoxicam (LOR) was also encapsulated in bilosomes for enhanced transdermal permeability. The in vivo pharmacodynamic activity in male rats and a mice model demonstrated enhanced anti-inflammatory and antinociceptive activity compared to that of the oral marketed product [66]. Recently, magnetic-targeted superparamagnetic iron oxide nanoparticles (SPIONS) loaded with LOR-bilosomes were developed for local intramuscular administration in osteoarthritis [67]. El-Nabarawi et al. [68] prepared and characterized dapsone (DPS)-loaded bilosomes for the topical treatment of acne, in which the optimized DPS-loaded bilosomes showed 170.57 ± 55.12 µg/mL DPS retention compared to 120.24 ± 10.7 µg/mL from DPS alcoholic solution in an ex vivo deposition study.

Abdelalim et al. [69] formulated nanobilosomes integrated with the sodium tauroglycocholate (STGC) of poorly bioavailable sildenafil citrate (SC) for topical delivery to treat erectile dysfunction. An ex vivo permeability study using human skin showed improved permeation and about 39% of the drug permeated within 15 min. An in vivo study in Sprague Dawley rats also revealed a 2-fold increase in intromission frequency and intromission ratio compared to the untreated group. The study findings concluded that there was a fast onset of action using bilosomes as a topical drug delivery system for treatment of impotency at low dose, which is about 20% of the conventional dose [69]. In another study, Mosallam et al. [70] successfully transformed the antifungal medication terconazole (TCZ) into highly deformable bilosomes (HBs) for topical administration, despite its limited permeability. HBs significantly reduced the Candida albicans growth compared to TCZ suspension and were evaluated using XTT (2,3-bis(2-methyloxy-4-nitro-5-sulfophenyl)-2H-tetrazolium-5-carboxanilide) reduction assay. In vivo studies showed an enhanced improvement in TCZ skin deposition compared to traditional bilosomal formulation and TCZ suspension [70]. Mahmoud et al. [71] developed topical diclofenac sodium (DNa)-loaded bilosomal gel for treating inflammation. The oral administration of DNa is associated with GI side effects. The increased efficacy of bilosomal formulation was confirmed by performing ex vivo permeation studies showing 2.5 times higher skin permeability than the DNa solution. In vivo studies in the paw edema rat model also revealed a significant suppression of inflammatory mediators and a reduction in paw edema, thereby demonstrating the greater efficacy of the bilosomal system in transdermal drug delivery [71]. Salem et al. [72] investigated the possible use of bilosomes as a carrier for the active targeted transdermal distribution of metformin hydrochloride, with the aim of mitigating its adverse effects. The release profile of the optimized formulation within 24 h was found to be 75.28% and permeation flux was found to be enhanced (198.79–431.91 ng cm^−2^ h^−1^) in comparison to the plain drug (154.26 ng cm^−2^ h^−1^). These findings indicate that the incorporation of metformin hydrochloride, an antidiabetic drug, into bilosomes resulted in enhanced permeation and provided a novel nanocarrier approach for effective transdermal administration [72]. Another antifungal drug miconazole nitrate (MN), which is poorly water-soluble, was loaded into chitosan–carbopol bilosomal gel to improve its therapeutic efficacy through a transdermal route [73]. Khafagy et al. [74] developed and evaluated topical novel simvastatin (SMV)-loaded bilosomal gel for treating inflammation. A 3-fold enhancement in SMV transdermal flux was shown by SMV-loaded bilosomal gel compared to the suspension of the pure drug [74]. Another poor orally bioavailable drug, dronedarone hydrochloride (DRN), was also formulated into bilosomal nanogel to enhance transdermal permeability and bioavailability. The optimized bilosomal gel formula demonstrated an in vitro drug release of 57.0 ± 8.68% relative to 13.3 ± 1.2% from drug suspension after 12 h. The ex vivo permeation study findings showed an enhanced skin permeation of 21.23 ± 1.54 µg/cm^2^ h across rat skin and collectively showed the superior efficacy of bilosomal gel compared to free drug suspension [75].

The process of delivering drugs through the skin is challenging because of several barriers that restrict permeation. This necessitates the use of external agents or carrier systems to facilitate successful penetration, which has led to the emergence of bilosome vesicular systems as a novel approach to therapy. The use of bilosomes for the topical or transdermal delivery of drugs is beneficial as it can avoid the first-pass metabolism and can lead to increased bioavailability. Moreover, as per currently reported studies, the histological examination of bilosomes also ensures the safety and tolerability of this vesicular system. Because of this, formulation scientists are now exploring the topical and transdermal delivery of active pharmaceutical components for their superior efficacy as an alternative to the oral administration of medications.

**Table 1:** Current perspectives on bilosomes as transdermal drug delivery system.

No.	Drug	Formulation	Therapeutic Problem	Study Outcomes	Ref.
1	Diacerin (DCN)	Bilosomes	Poorly bioavailable (35–56%) osteoarthritic drug with gastrointestinal side effects on oral administration	Bilosomes were found to be superior than niosomes and pure suspension for enhancing DCN flow through the skin	[11]
2	Olmesartan medoxomil (OLM)	PEGylated bilosomes	Poor oral bioavailability of 26% which limits its therapeutic efficacy	Higher OLM deposition in rat’s skin from bilosomal formulation compared to transethosomes and OLM suspension was found as per in vivo skin deposition studies	[24]
3	Tizanidine HCL (TZN)	Bilosomes	Poor bioavailability	Enhanced transdermal permeation compared to plain drug	[64]
4	Terbutaline sulfate (TBN)	TBN chitosan-coated bilosomes (TBN-CTS-BLS)	Poor oral bioavailability due to hepatic first-pass metabolism	2.33-fold increase in TBN bioavailability and t_1/2_ was increased to 6.21 ± 0.24 h when using the optimized TBN-CTS-BLS formulation compared to the oral TBN solution in the rat model	[65]
5	Lornoxicam	Bilosomes	Poor aqueous solubility and rapid clearance, GIT toxicity	In vivo pharmacodynamic activity in male rats and mice model demonstrated enhanced anti-inflammatory and antinociceptive activity compared to that of the oral marketed product	[66]
6	Dapsone (DPS)	Bilosomes	Low solubility and toxicity through oral administration	Ex vivo deposition study of optimized DPS-loaded bilosomes showed 170.57 ± 55.12 μg/mL DPS retention compared to 120.24 ± 10.7 μg/mL from DPS alcoholic solution	[68]
7	Sildenafil citrate (SC)	Nanobilosomes	Poor bioavailability	In vivo study in Sprague Dawley rats revealed 2-folds increase in intromission frequency and intromission ratio compared to untreated group	[69]
8	Teroconazole (TCZ)	Highly deformable bilosomes (HBs)	Poor permeability	In vivo studies showed improvement in terconazole skin deposition compared to typical bilosomal formulation and TCZ suspension	[70]
9	Diclofenac sodium (DNa)	Bilosomal gel	Gastrointestinal side effects on oral administration	Ex vivo permeation studies of optimized formulation showed a 2.5-fold increase in skin permeability than DNa solution, In vivo studies in paw edema rat model also revealed significant suppression of inflammatory mediators and reduction in paw edema	[71]
10	Metformin HCL	Bilosomes	Adverse effects	Permeation flux was found to be enhanced (198.79–431.91 ng cm ^−2^ h^−1^) in comparison to the plain drug (154.26 ng cm ^−2^ h^−1^).	[72]
11	Miconaole nitrate (MN)	Chitosan–carbopol bilosomal gel	Poor water solubility	Enhanced antifungal activity compared to pure drug against *Candida albicans* and *Aspergillus niger*	[73]
12	Simvastatin (SMV)	Bilosomal gel	Poor bioavailability due to water insolubility and hepatic first-pass effect.	3-fold increase in SMV transdermal flux from SMV-loaded bilosomal gel compared to plain drug suspension. Bioavailability of SMV-BS gel was also found to be ~2-fold and ~3-fold higher than those of oral SMV suspension and SMV gel, respectively	[74]
13	Dronedarone hydrochloride (DRN)	Bilosomal nanogel	Poor bioavailability	Bilosomal gel demonstrated enhanced release (57.0 ± 8.68% of DRN compared to only 13.3 ± 1.2% released from drug suspension after 12 h) and enhanced skin permeation	[75]

### 4.6. Bilosomes as Cancer Drug Delivery System

The global rise in cancer mortality rates has prompted an intensified pursuit of innovative treatment interventions. Bilosomes have numerous advantages over other lipid-based vesicular systems for the delivery of anticancer drugs such as GIT stability, superior intestinal permeation, being more deformable, and possessing increased drug loading. Consequently, a range of vesicular delivery methods are being used for the targeted administration of medicines to tumor cells. There is ongoing research on the potential of bilosomes for delivering drugs for cancer treatment [76].

The delivery potential of many anticancer drugs using bilosomes as a nanocarrier was investigated by numerous scientists as shown in Table 2. Cytostatic bile acid derivatives, Bamet-R2 [cis-diamminechloro cholylglycinate platinum(II)] and Bamet-UD2 (cis-diammine bis-ursodeoxycholate platinum(II) have been studied for their potential to inhibit cell proliferation in a variety of hepatocellular cancer cell lines, including LS174T/R (human colon adenocarcinoma), WIF-B9/R (rat hepatoma–human fibroblast hybrid), and Hepa 1-6/R (mouse hepatoma). The absorption and cytostatic activity of Bamets were much greater than the poorly water-soluble anticancer drug cisplatin in their wild-type counterparts. Bamets were effective in penetrating all cell lines that had developed resistance to cisplatin. One study reported the incorporation of these Bamets in the liposomes and demonstrated their potential for overcoming chemotherapy resistance, especially in cases with enterohepatic circuit tumors [77]. Parashar et al. [78] developed bilosomes that were modified with dextrose (DEX) and loaded with silymarin (DEX-SYL-BL) for peroral and targeted drug delivery for treating hepatic carcinoma. The therapeutic efficacy of silymarin (SYL) is limited due to its inadequate bioavailability and low solubility. Cytotoxicity studies performed in Hep-G2 cell lines showed a greater reduction in cell viability using DEX-SYL-BL when compared with pure SYL and SYL-containing bilosomes (SYL-BL). In vivo results evaluated in a mice model of diethyl nitrosamine (DEN)-induced hepatic carcinoma showed that DEX-SYL-BL treated mice exhibited an improved lifespan and a reduced tumor burden, indicating a greater therapeutic potential of the bilosomes in anticancer drug delivery [78]. Zaki et al. [79] synthesized acrylamide derivatives and evaluated their cytotoxicity against human breast cancer MCF-7 cells. Compound **4e**, which was found to have more cytotoxicity potential against MCF-7 cells lines, was further loaded into PEGylated bilosomal nanovesicles in order to enhance the suboptimal oral bioavailability. A remarkable improvement in the cytotoxic activity of the compound **4e** from PEGylated nanovesicle (IC_50_ = 0.75 ± 0.03 µM) was found compared to the pure form (IC_50_ = 2.11 ± 0.19 µM) [79]. In another study, Alhakamy et al. [80] developed and evaluated icariin-loaded bilosomes-melittin (ICA-BM) in an attempt to increase the efficacy of these natural biomolecules against pancreatic cancer cells, PNAC1. The evaluation of pro-apoptotic protein levels, cell cycle analysis, and annexin V staining in PNAC1 cells demonstrated that the optimized ICA-BM formulation significantly augmented icariin’s efficacy in combating malignant pancreatic cells [80]. Another study by the same research group evaluated piceatannol (PIC), an herbal polyphenolic compound with anti-cancercytotoxic activity, which was loaded into zein-modified bilosomes for treating lung cancer. In this study, the cytotoxicity evaluation of the optimized formulation revealed smaller IC_50_ against A549 cells compared to the pure drug [81]. Zafar et al. [82] developed luteolin-loaded PEGylated bilosomes (LL-BLs) for oral administration. Human breast cancer cell lines, MDA-MB-231 and MCF-7, were used in the cytotoxicity study. Luteolin-loaded pegylated bilosomes showed a greater cell viability than pure drug on both cell lines. It was found that the IC_50_ values for MCF-7 and MDA-MB-231 cancer cells were 390 M and 510 M, respectively. Therefore, PG-BLs may offer an effective nano-oral delivery system for specifically targeting a breast cancer cell line [82]. Matloub et al. [83] delivered sulfated polysaccharide–protein complexes that had a significant anticancer potential extracted from Enteromorpha intestinalis (EHEM), using bilosomes as a delivery carrier in order to treat hepatocellular carcinoma. Based on the in vivo results, an optimized bilosomal formulation can be employed as a prospective option for treating hepatocellular carcinoma due to its strong anti-cancer and anti-angiogenic action [83].

Curcumin (CUR), a naturally occurring polyphenolic phytoingredient, possesses strong anti-inflammatory, antioxidant, and anticancer properties, but its clinical efficacy is limited due to poor oral bioavailability and permeability [84]. Abbas et al. [85] developed nano-bilosomes for the delivery of the CUR analogue, 3,5-bis(4-bromobenzylidene)-1-propanoylpiperidin-4-one, in order to enhance its bioavailability. The antitumor selectivity index of CUR analogue-loaded bilosomes was reported to be significantly higher against Huh-7 liver cancer cells as compared to a CUR suspension or doxorubicin, another standard anticancer drug [85]. Hegazy et al. [86] also designed D-alpha-tocopheryl polyethylene glycol succinate (TPGS)-coated bilosomes for the oral delivery of CUR for enhanced antitumor activity. In a Caco-2 cell lines cellular uptake study, 61.9 ± 5.3% uptake of CUR was demonstrated from TPGS-CUR-bilosomes compared to 7.4 ± 2.12% from CUR suspension. Ex vivo permeation was also found to be increased by 6.6 folds from TPGS-CUR-bilosomes compared to pure suspension [86]. So, the therapeutic efficacy of CUR in terms of solubility, permeability, and stability is found to be improved significantly by using bilosomes as a nano-delivery carrier.

Imam et al. [87] loaded apigenin into chitosan-coated bilosomes to enhance its bioavailability. The antimicrobial and cytotoxicity analysis in two distinct cancer cell lines, MCF-7 breast cancer cells, and A549 lung cancer cells, showed better activity than pure apigenin. Pitavastatin (PIT) has been demonstrated to provide an anticancer effect against hepatocellular carcinoma (HCC) in addition to its well-known efficacy as an anti-hyperlipidemic drug. Kharouba et al. [88] developed and evaluated PIT-loaded bilosomes for oral delivery in treating HCC. In this study, the surface modification of bilosomes with lactoferrin was also evaluated and the results showed an improvement in the cytotoxicity of HepG2 spheroids, which was 44 times greater than that of PIT solution. Moreover, the apoptotic potential of PIT was found to be increased by two-fold, suggesting the potential application of bilosomes as a drug delivery system for treating cancer [88]. Another bioactive compound quercetin (QT), which is reported to possess antioxidant and chemoprotective activity, suffers from a poor bioavailability problem. To improve its therapeutic efficacy, Alruwaili et al. [89] developed surface-modified bilosomes loaded with QT. The results demonstrated 1.61-fold higher cytotoxicity of surface-modified chitosan-coated QT-bilosomes against MFC-7 and 1.44-fold higher cytotoxicity against MDA-MB-231 breast cancer cells than pure QT.

The oral bioavailability and antitumor activity of psoralidin (Ps), which also possess strong antimicrobial, antioxidant, antipsoriatic, anti-inflammatory, and antidepressant activities [90], is improved by developing its chitosan-coated bilosomes nanoformulation for oral delivery. The apoptotic and necrotic potential of the developed formulation was evaluated in MCF-7 human breast cancer cells and A549 human lung cancer cells A549, in which a significant increase in the percentage of the apoptotic and necrotic cells were found compared to the control and free Ps [91]. Doxorubicin (DOX) is another potent anticancer drug whose clinical applicability is limited due to dose-related toxicity. DOX was loaded into bilosomes for improved cytotoxic activity and systemic absorption [18]. The investigations of the optimized formulation showed an improved DOX cytotoxicity against MCF-7 breast cancer cells and a reduced IC_50_ value from 13.3 μM to 0.1 μM. A 4.5–6 and 1.8–2.5-fold increase in drug absorption was reported from the jejuno-ileum and colon, respectively [18].

Scientists have successfully tested diverse molecular and vesicular methodologies for the therapeutic delivery of several anticancer drugs. Bilosomes have a significant relevance in this context due to their capacity to enhance several aspects of cytotoxic efficacy against cancerous cells. The potential of stimuli-sensitive bilosomes as a vesicular carrier in cancer cell targeting still needs to be explored, as there is very limited literature available currently.

**Table 2:** Reported bilosomal formulation for cancer drugs.

No.	Drug	Formulation	Therapeutic Problem	Study Outcomes	Ref.
1	Cisplatin	Cytostatic bile acid incorporated liposomes	Cisplatin resistance in cancer cell lines	Enhanced ability of anticancer drug to be taken up by cancer cell due to the presence of cytostatic bile acids.	[77]
2	Silymarin	Dextrose-modified bilosomes	Poor bioavailability and low solubility	Dextrose bilosomes loaded with silymarin were tested in vivo for treating Diethyl nitrosamine (DEN)-induced hepatic malignancy. The mice had longer lifespans and less tumor load, suggesting more therapeutic potential.	[78]
3	Lead acrylamide molecules	Lead acrylamide molecules utilizing **4e**-charged PEGylated bilosomes	Poor bioavailability and low solubility	Cytotoxicity testing showed that compounds **4e** and **5d** were effective against MCF-7 cells. After integration into the nano-PEGylated bilosomal system, the drug’s cytotoxic activity was increased.	[79]
4	Icariin and Melittin	Icariin-loaded bilosomes-melittin (ICA-BM)	Poor bioavailability and low solubility	ICA-BM showed a lower IC_50_ than blank-BM and ICA-pure. ICA-BM formulation increased icariin’s effectiveness against malignant pancreatic cells.	[80]
5	Luteolin	PEGylated bilosomes (LL-BLs)	Poor bioavailability and low solubility	LL-BLs showed greater cell viability than the pure drug on MDA-MB-231 and MCF-7 breast cancer cell lines. It was found that the IC_50_ values for MCF-7 and MDA-MB-231 cancer cells were 390 M and 510 M, respectively.	[82]
6	Sulfated polysaccharide–protein complexes	Bilosomes	Poor bioavailability and low solubility	Substantial decrease in serum α-fetoprotein, endoglin, lipocalin-2, and heat shock protein 70 levels. The photomicrographs of rat liver tissue slices showed a focal area of pleomorphic hepatocytes that had deteriorated, together with fine fibrosis emanating from the portal region.	[83]
7	Curcumin (CUR) analogue 3,5-bis(4-bromobenzylidene)-1-propanoylpiperidin-4-one	Nano-bilosomes	Poor bioavailability and permeability	Antitumor selectivity index of CUR analogue-loaded bilosomes recorded 420.55 against liver cancer cells when compared to a CUR suspension.	[85]
8	Apigenin	Chitosan-coated bilosomes	Poor bioavailability and low solubility	Antimicrobial and cell viability analyses demonstrated better outcomes with regard to inhibition and cell line assessment against two MCF-7 breast cancer and A549 lung cancer cell lines.	[87]
9	Pitvaststin	Bilosomes	Poor bioavailability	Improvement in the cytotoxicity of HepG2 spheroids, which was 44 times greater than that of pitavastatin (PIT) solution.	[88]
10	Quercetin	Chitosan-coated quercetin-bilosomes	Low bioavailability	1.61-fold higher cytotoxicity of surface-modified chitosan-coated quercetin-bilosomes against MFC7 and 1.44-fold higher cytotoxicity against MDA-MB-231 than pure quercetin.	[89]
11	Psoralidin (Ps)	Chitosan-coated bilosomes	Water insoluble, dose-dependent toxicity	Apoptotic and necrotic potential of the developed formulation was evaluated in human breast cancer cell lines (MCF-7) and human lung adenocarcinoma cell lines (A549), in which a significant increase in the percentages of the apoptotic and necrotic cells was found compared to the control and free Ps.	[91]
12	Doxorubicin (DOX)	Bilosomes	High dose-related toxicity	Formulation showed improved DOX cytotoxicity against breast cancer cells (MCF-7) and the reduced IC_50_ value from 13.3 μM to 0.1 μM. A 4.5–6 and 1.8–2.5-fold increase in drug absorption from jejuno-ileum and colon was demonstrated.	[18]

### 4.7. Bilosomes for Delivery of Herbal Drugs

Several alternative medicinal practices have been widely acknowledged and consistently used across the world, which include Ayurveda, Yoga, Unani, Siddha, Homeopathy, and Naturopathy. The herbal medicine industry has significant potential for expansion. The significant demand for herbal medications in both developed and developing nations may be attributed to their diversified biological activity, relatively cheaper cost, and superior safety margin compared to synthetic therapies [92]. It has been observed that bilosomes had the potential to serve as a viable vehicle for improving the pharmacokinetic profile of herbal medications, as shown in Table 3.

Silymarin (SM), a polyphenolic flavonoid, is a strong hepatoprotective compound but its therapeutic use is limited due to poor water solubility and stability [93]. Mohsen et al. [12] developed and characterized SM-loaded bilosomes to enhance the drug’s hepatoprotective effects. Studies conducted in animals showed that SM-loaded bilosomes and liposomes had a stronger hepatoprotective effect than the drug alone. However, an ex vivo intestinal uptake study revealed the superiority of bilosomes compared to liposomes suggesting the potential application of bilosomes for delivering hepatoprotectants orally [12]. A combination of berberine (BER) CUR has been known for treating non-alcohol fatty liver disease (NAFLD). However, the synergistic effects of BER and CUR in combination are still a challenge to achieve due to their inconsistent bioavailability and biodistribution. To improve the synergistic ameliorative effects of these two natural compounds, Chen et al. [15] developed dextran-coated bilosomes-encapsulating BER and CUR for treating NAFLD. The in vivo pharmacokinetic evaluation of a developed bilosomal formulation in a mice model demonstrated improved oral absorption, synchronized biodistribution, and a prolonged circulation of both BER and CUR [15]. It can be stated that bilosomes are a promising drug delivery strategy for administrating herbal drug combinations orally with synchronized and enhanced efficacy against NAFLD. Moreover, stimuli-responsive self-assembled bilosomes loaded with CUR as a model compound and methylene blue dye were developed and investigated. One study reported a pH- and temperature-dependent release of the model compound from bilosomes [94]. In another study, for the treatment of diabetic (type-2) diabetes, Zafar et al. [95] developed bilosomes that were loaded with apigenin (AG), a natural flavone compound having anticancer and antidiabetic potential that suffers from poor aqueous solubility. In vivo permeation and pharmacokinetic studies showed a 4.49 times higher flux and a 4.67-fold higher AUC_0–t_ for the bilosomal formulation compared to the free compound [95]. Similarly, to boost piperine’s water solubility and its therapeutic efficacy, Zakaria et al. [96] developed piperine-loaded bilosomes. An in vivo pharmacokinetic study showed a higher oral bioavailability of piperine-loaded bilosomes than piperine suspension. Additionally, the antiviral activity and safety margin of an optimized bilosomal formulation were much higher than those of the drug suspension [96].

BER, a naturally obtained isoquinoline alkaloid, is reported to have potential anti-inflammatory, antidiabetic, and anticancer activities [97]. Its therapeutic efficacy is still a challenge because of poor aqueous solubility and permeability [98]. Many attempts have been made to enhance its bioavailability and efficacy. Elkomy et al. [16] encapsulated BER, a phytoconstituent used to treat chronic inflammation in rheumatoid arthritis, into chitosan-coated bilosomal gel to improve its transdermal delivery. An in vivo evaluation was performed in a rat model of carrageenan-induced paw edema which demonstrated a 24.4% edema swelling reduction after 12 h treatment [16]. In another work, Elkomy et al. [99] developed BER-loaded bilosomes to improve the oral bioavailability and therapeutic efficacy of BER for treating diabetes. In comparison to the BER solution, the bilosomal formulation showed greater stability and better-sustained release of BER for up to 8 h. An in vivo evaluation study for the optimized formulation showed a significant reduction in blood sugar, with a maximum drop of 41%, compared to the BER solution with a 19% reduction in sugar level [99]. Conclusively, the therapeutical efficacy of BER can be significantly improved using bilosomes as a nanocarrier delivery system. Quercetin (QT), a flavonoid with antioxidant and chemo-preventive properties, was encapsulated in surface-modified bilosomes by Alruwaili et al. [89]. The surface modification was performed with chitosan and surface-modified bilosomes-encapsulating QT, which showed remarkable antioxidant properties compared to pure QT. Likewise, it showed a 1.44-fold higher cytotoxicity relative to pure QT when tested against the MDA-MB-231 cell line [89]. These findings revealed that the efficacy of QT can be enhanced by encapsulating them in surface-modified bilosomes. Resveratrol (RSV), a phenolic compound with poor bioavailability, was loaded into nanobilosomes for increased cell permeability and antiviral activity [100]. An in vitro drug release study demonstrated an 82.4 ± 3.8% extended drug release for over 12 h compared to 21.9 ± 1.9% from RSV suspension. In vitro studies on Caco-2 cells showed a 4.7-fold increase in cellular uptake as compared to RSV dispersion [100].

Conclusively, it can be stated that bilosomes are effective nanovesicles for delivering herbal compounds through an oral and transdermal route of administration. Bilosomes are reported to be a new generation of nanovesicles which are superior to liposomes and niosomes for the therapeutic delivery of natural drugs due to their stability, deformable flexibility, and permeability characteristics. The integration of Ayurveda, contemporary medicine, and scientific methodologies is a formidable catalyst for exploration, potentially yielding the development of enhanced therapeutic interventions that are both safer and more cost-effective while minimizing adverse effects. This is attributed to the ability of bilosomes to shield the pharmaceuticals from harsh GI conditions, hence impeding the detrimental impact of stomach acid and enzymes.

**Table 3:** Current perspectives on bilosomes incorporating herbal drugs.

No	Drug	Formulation	Therapeutic Problem	Outcome of Study	Ref.
1	Silymarin	Bilosomes	Poor water solubility and stability	Ex vivo intestinal uptake study revealed the superiority of bilosomes compared to liposomes for strong hepatoprotective effect	[12]
2	Berberine (BER) and curcumin (CUR)	Bilosomes	Poor bioavailability and biodistribution	In vivo pharmacokinetic evaluation of bilosomal formulation in mice model showed improved and synchronized oral bioabsorption of both BER and CUR	[15]
3	Apigenin (AG)	Bilosomes	Poor bioavailability and low solubility	In vivo permeation and pharmacokinetic studies showed that the free AG-dispersion had a 4.49 times higher flux and a 4.67 folds higher AUC_0–t_	[95]
4	Piperine	Bilosomes	Poor bioavailability and low solubility	Piperine-loaded bilosomes had higher oral bioavailability than piperine suspension. Additionally, the optimized formula’s antiviral activity and safety margin were much higher than those of the drug suspension	[96]
5	Berberine (BER)	Chitosan-coated bilosomal gel	Poor bioavailability and low solubility	In comparison to the BER solution, formulation showed greater stability and sustained release of BER. In vivo study, formulation significantly reduced blood sugar, with a maximum drop of 41%, compared to BER-blood SOL’s sugar reduction of only 19%	[16]
6	Resveratrol (RSV)	Nanobilosomes	Poor bioavailability	Caco-2 cell lines study showed 4.7 fold increase in cellular uptake compared to RSV dispersion	[100]

## 5. Conclusions and Future Perspectives

Bilosomes can encapsulate proteins, peptides, anticancer compounds, phytoconstituents, and several other therapeutic compounds that exhibit poor water solubility and limited bioavailability. Based on the findings of the literature search, it has been observed that the bilosomes have the potential to enhance the therapeutic efficacy and bioavailability of certain substances as they are deformable nanovesicles with enhanced permeation properties due to the presence of bile salts. In addition, bilosomes that are engineered with surface modifications have shown the ability to selectively target the therapeutic agents to specific cells or organs through the attachment of ligands onto the bilosomal surface. Bile acids have garnered growing attention as valuable constituents for the development of novel medicines and drug delivery systems due to their convenient accessibility, cost-effectiveness, and straightforward derivatization procedures.

The current need is to direct research efforts toward investigating the targeted transportation of drugs to the intestinal lymphatic system, specifically at the cellular level, using bilosomes. The investigation of the potential use of bilosomes for conveying a diverse array of antigens with varying physicochemical properties and a susceptibility to degradation in the gastrointestinal tract is necessary. The use of an intelligent bilosomal drug delivery strategy is anticipated to enhance the efficacy against many life-threatening illnesses by targeting the lymphatic system. Additionally, there is a lack of research papers on stimuli-sensitive bilosomes for drug delivery. It is indeed an area that warrants further exploration and study. The development of stimuli-sensitive bilosomes holds great potential for enhancing drug delivery efficiency, especially in targeting specific tissues or cancer cells with controlled release properties. By exploring these unique characteristics, researchers can uncover new opportunities for improving drug delivery systems and addressing challenges in various fields. Consequently, it is anticipated that bilosomes will play a vital role in combating and ultimately eradicating severe and contagious illnesses. Presently, it is of paramount significance for clinical researchers to use the expertise surrounding bilosomes to conduct safe and efficacious studies involving human patients, thus elucidating the precise mechanism behind the administration of bilosomes.
